# Supplementary figures and images for: Saturation genome editing of 11 codons and exon 13 of BRCA2 coupled with chemotherapeutic drug response accurately determines pathogenicity of variants
Source: PLoS Genet. 2023 Sep 15;19(9):e1010940. doi: 10.1371/journal.pgen.1010940 (PMC10529611; doi:10.1371/journal.pgen.1010940)

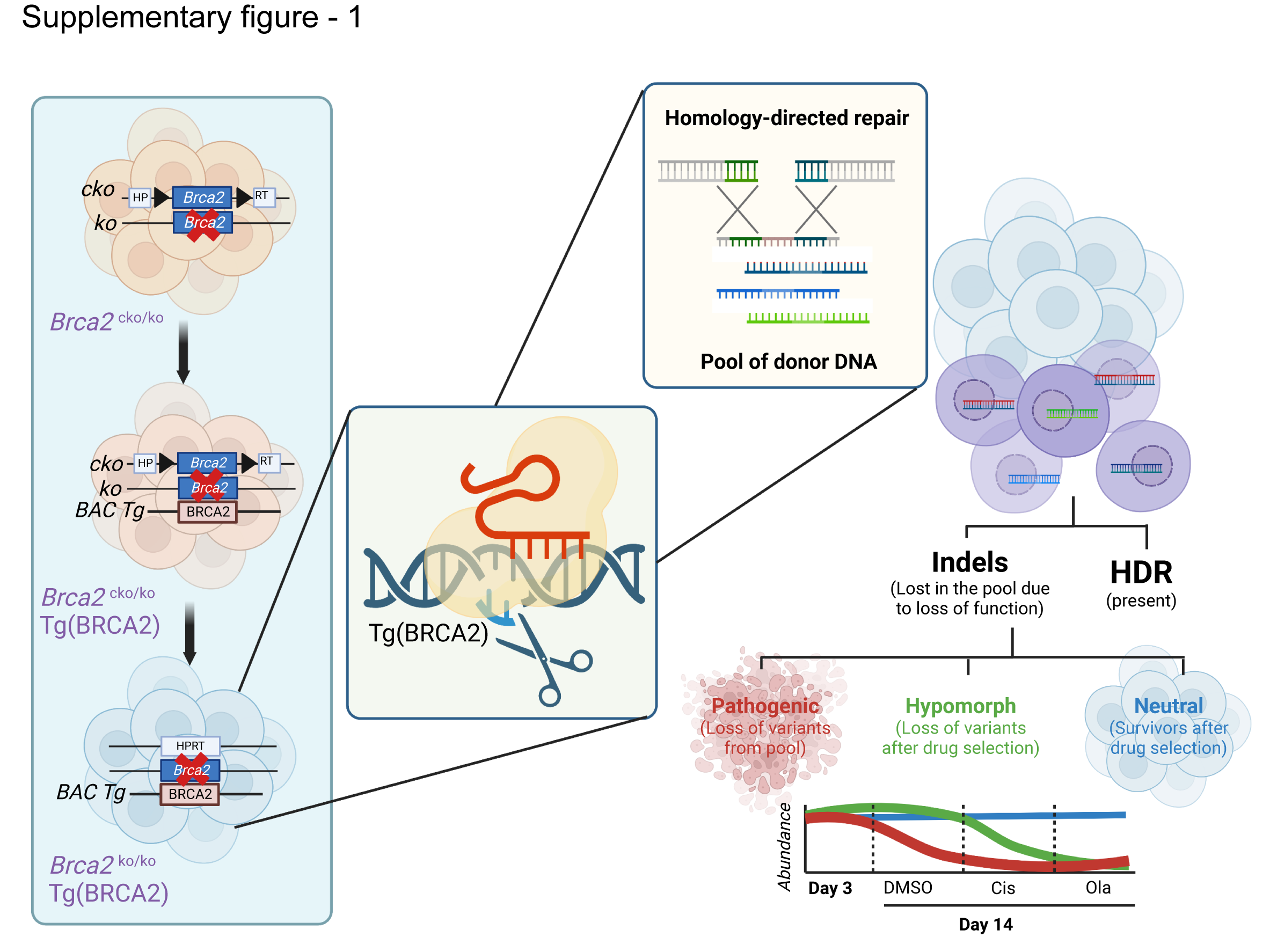

Supplement: S1 Fig — CRISPR-Cas9 based editing along with a pool of donor DNA containing NNN degenerate nucleotides, directly into the integrated BRCA2 transgene (Tg) allows generation of BRCA2 variants. The generation of indels is also limited as loss of function variants will be lost from the pool and only variants generated by HDR will survive. All HDR variants that are generated by CRISPR-Cas9 SGE will be present at the initial time point at day 3 and loss of function variants (pathogenic) will be eventually lost by day 14. Hypomorphic variants with partial loss of BRCA2 function are sensitive to DNA damaging drugs, cisplatin and PARP inhibitor (olaparib), thereby distinguishing between hypomorphic and neutral variants. Figure prepared using a paid subscription to BioRender.com. (TIF) [file pgen.1010940.s001.tif]

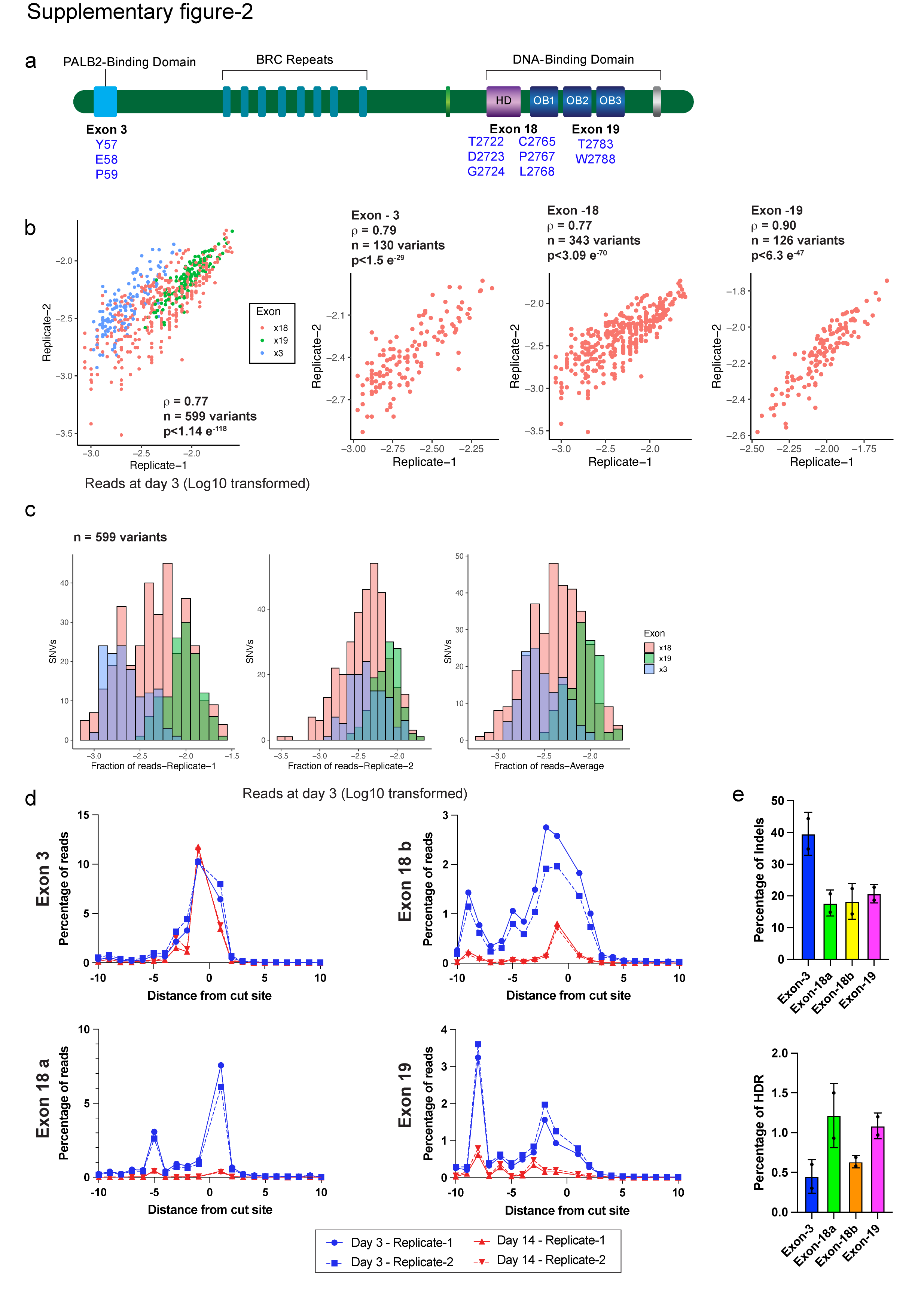

Supplement: S2 Fig — (a) Schematic representation of 3418 amino acid long BRCA2 showing its key functional domain. Variants were generated at residue 57, 58, 59 of Exon 3 and 2722, 2723, 2724, 2765, 2767, 2768 of exon 18 and 2783,2788 of exon19. (b) Pearson correlation showing the read abundance for each variant at day 3 between two independent replicates. The data is represented in log10 scale, ρ = 0.77 for 599 BRCA2 variants. Each exon is color-coded. The read counts were strongly correlated between individual exons (ρ = 0.79 for 130 variants of Exon3, ρ = 0.77 for 343 variants of Exon 18 and ρ = 0.90 for 126 variants of Exon 19) (c) Histogram showing the distribution of read abundance for each replicate and the average of the read abundance. (d) Graph representing the distribution of indels from the cut site “0” for each exon. The values were expressed as percentage of reads normalized to the total number of reads for each exon at day 3 and at day 14. Blue line represents day 3 and red line represents day 14. (e) Quantification showing the percentage of indels and percentage of HDR between individual exons. Each dot represents an independent replicate. (TIF) [file pgen.1010940.s002.tif]

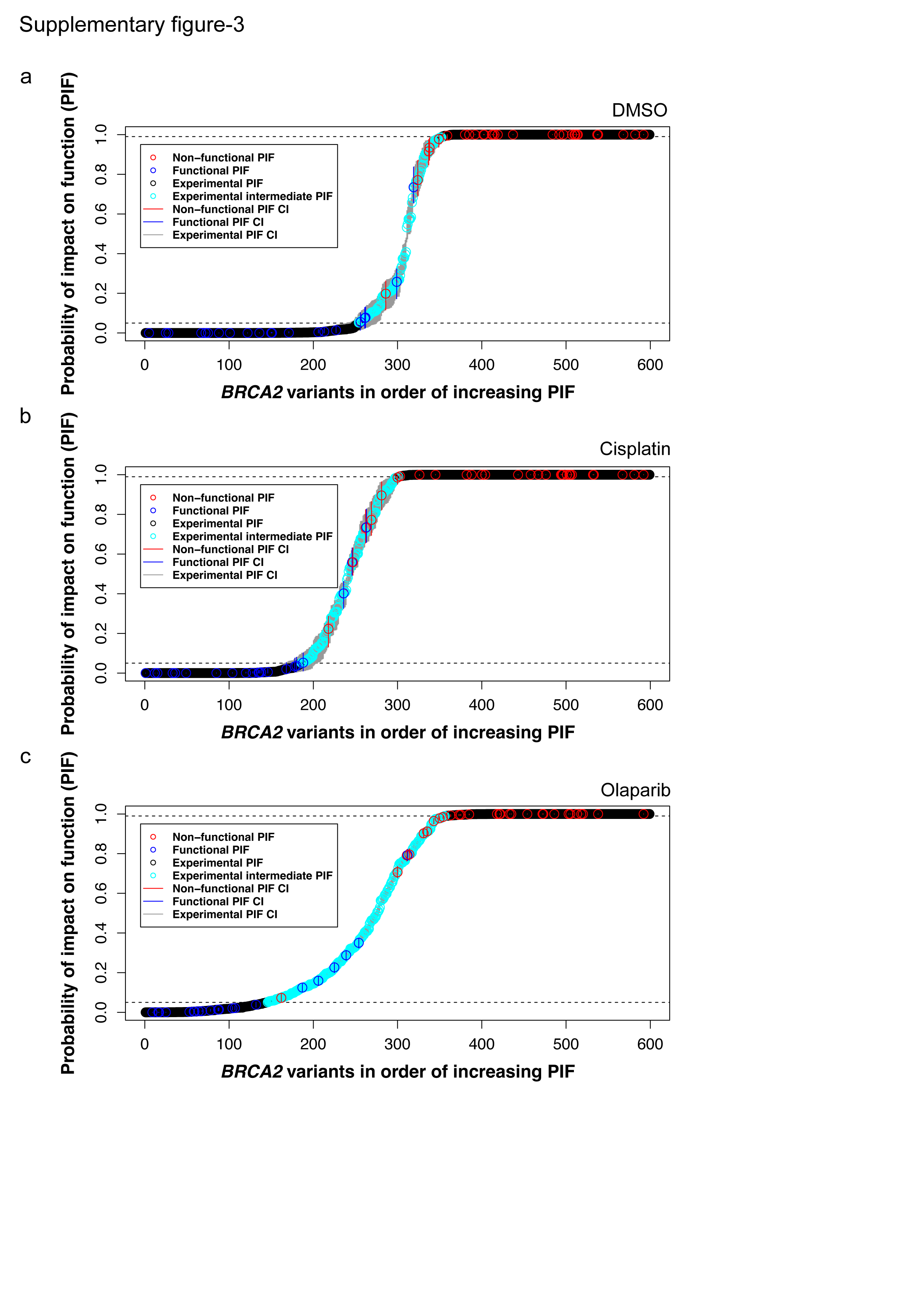

Supplement: S3 Fig — Probability of impact on function (PIF) for the BRCA2 variants in the full data set (N = 599), calculated using (a) only DMSO-assay data (b) only cisplatin-assay data (c) only olaparib-assay data. The circles represent individual BRCA2 variants, and the vertical lines show the 95% confidence intervals (CIs) for each PIF (for some of the variants, the confidence intervals are negligibly small). The dashed lines correspond to the functional-classification thresholds of 0.05 and 0.99. Most of the black circles are positioned so close that they form a continuous thick black line. (TIF) [file pgen.1010940.s003.tif]

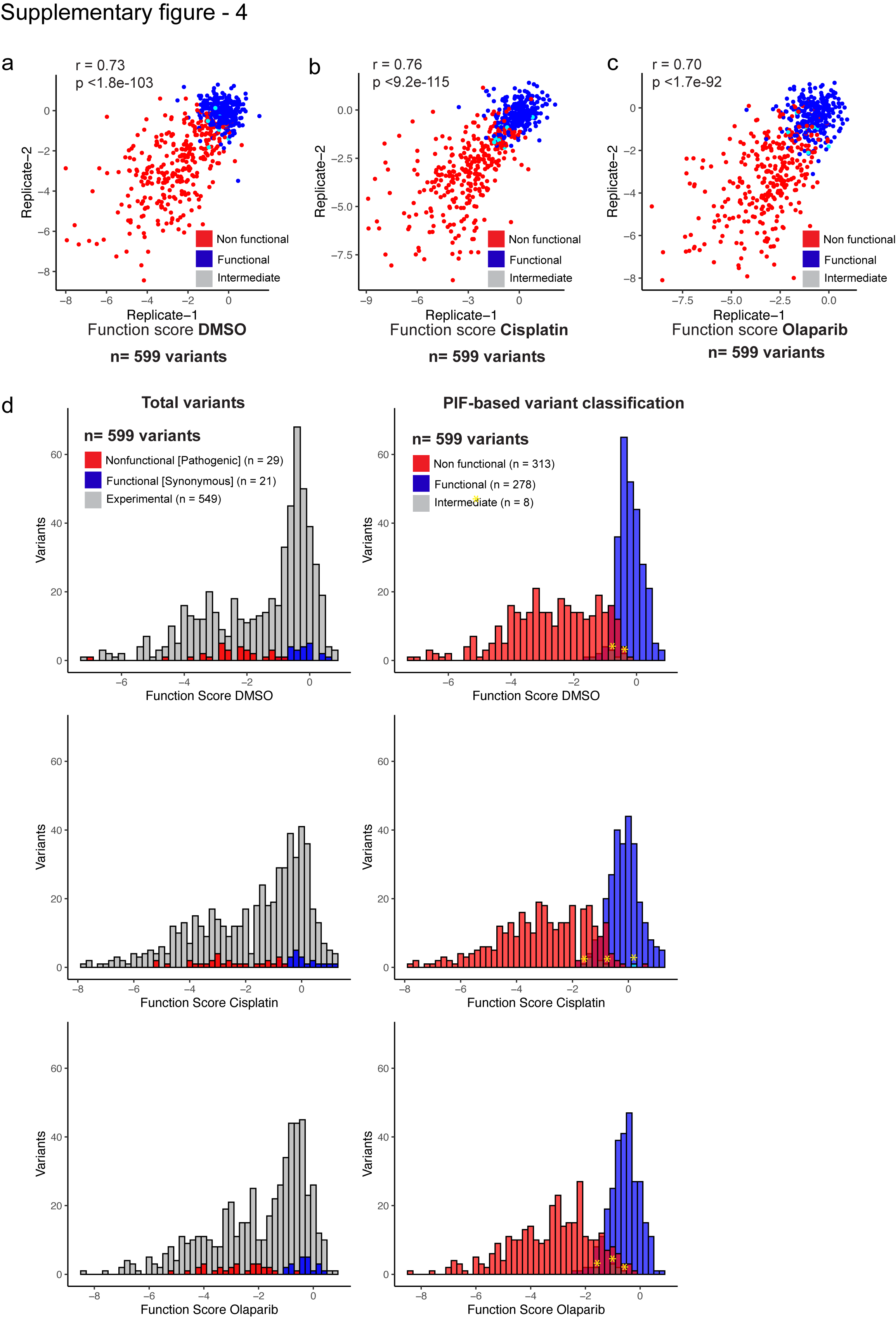

Supplement: S4 Fig — (a-c) A strong correlation of the function scores between two independent replicates for 599 variants (r>0.7) for DMSO, cisplatin and olaparib samples. Red dots represent nonfunctional, blue dot represents functional and gray dot represents intermediate category of variants. (d) Histogram showing the distribution of 599 variants of which 29 variants are known to be non-functional as it encodes for a stop codon, 21 variants are known to be functional as it leads to a synonymous change in nucleotide with no change in amino acid. Remaining 549 variants were experimental and are bimodally distributed in DMSO, cisplatin and olaparib samples. The known functional and nonfunctional variants were used as a training dataset to develop the statistical classifier. (e) The probability of impact on function (PIF) was calculated for all the 549 experimental variants. The histogram shows the distribution of PIF-classified functional (278 variants), nonfunctional (313 variants) and 8 variants that falls into the intermediate zone were denoted by an asterisk. (TIF) [file pgen.1010940.s004.tif]

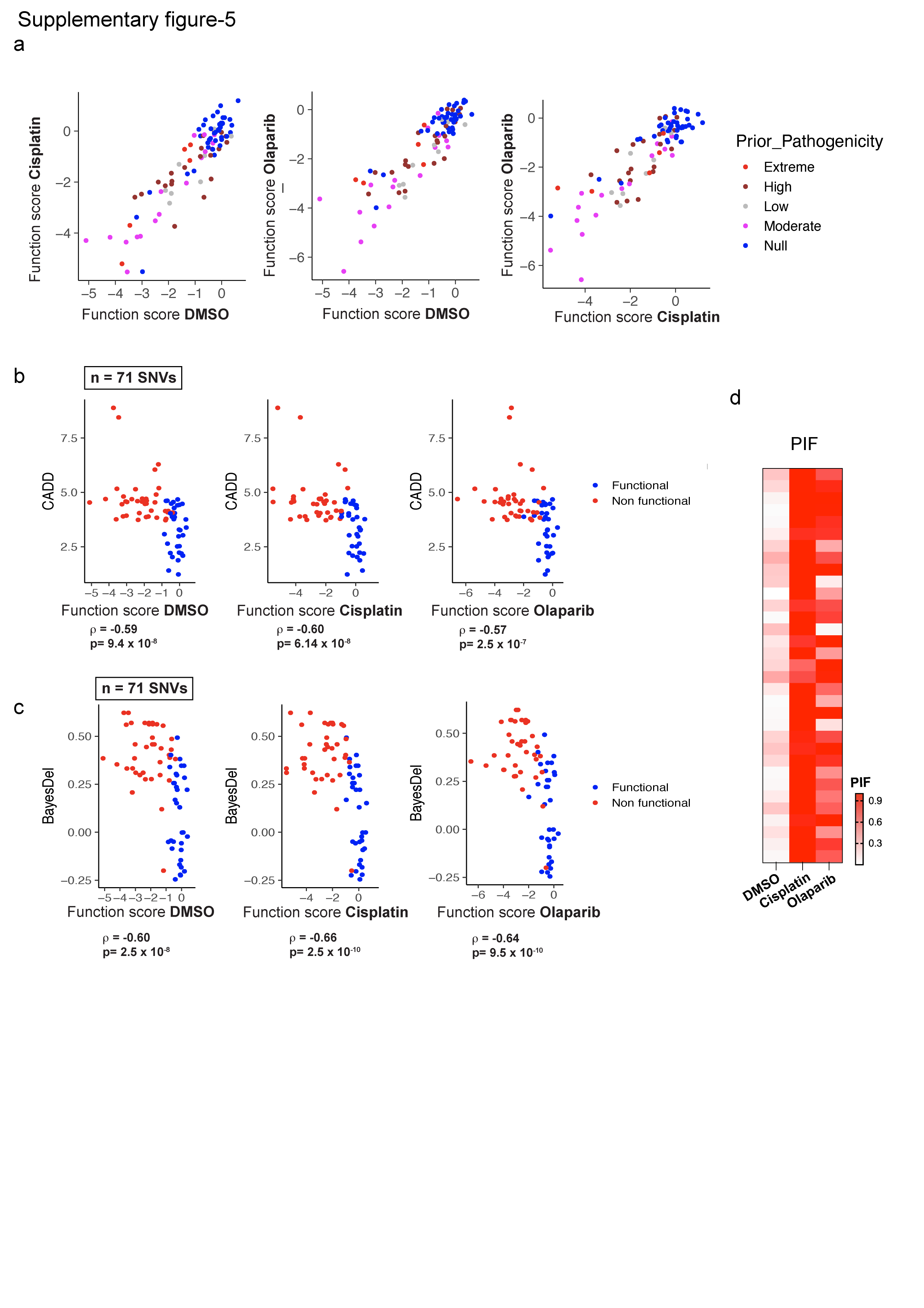

Supplement: S5 Fig — (a) Strong correlation of function scores between DMSO and cisplatin, DMSO and olaparib, cisplatin and olaparib samples showing the distribution of BRCA2 SNVs labelled based on PRIOR pathogenicity class. (b-c) Negative correlation between SGE-derived function scores of single nucleotide variants (SNVs) across 11 codons in DMSO, cisplatin and olaparib with (b) CADD scores (ρ = -0.57 to -0.60, n = 71 SNVs) and (d) Bayes-del score (ρ = -0.6 to -0.66, n = 71 SNVs). Blue dots represent the functional and red dots are non-functional variants according to our PIF-based calculation. (d) Heatmap showing the PIF score distribution of 33 variants that show functional PIF in DMSO but are sensitive to either or both the DNA damaging drugs. (TIF) [file pgen.1010940.s005.tif]

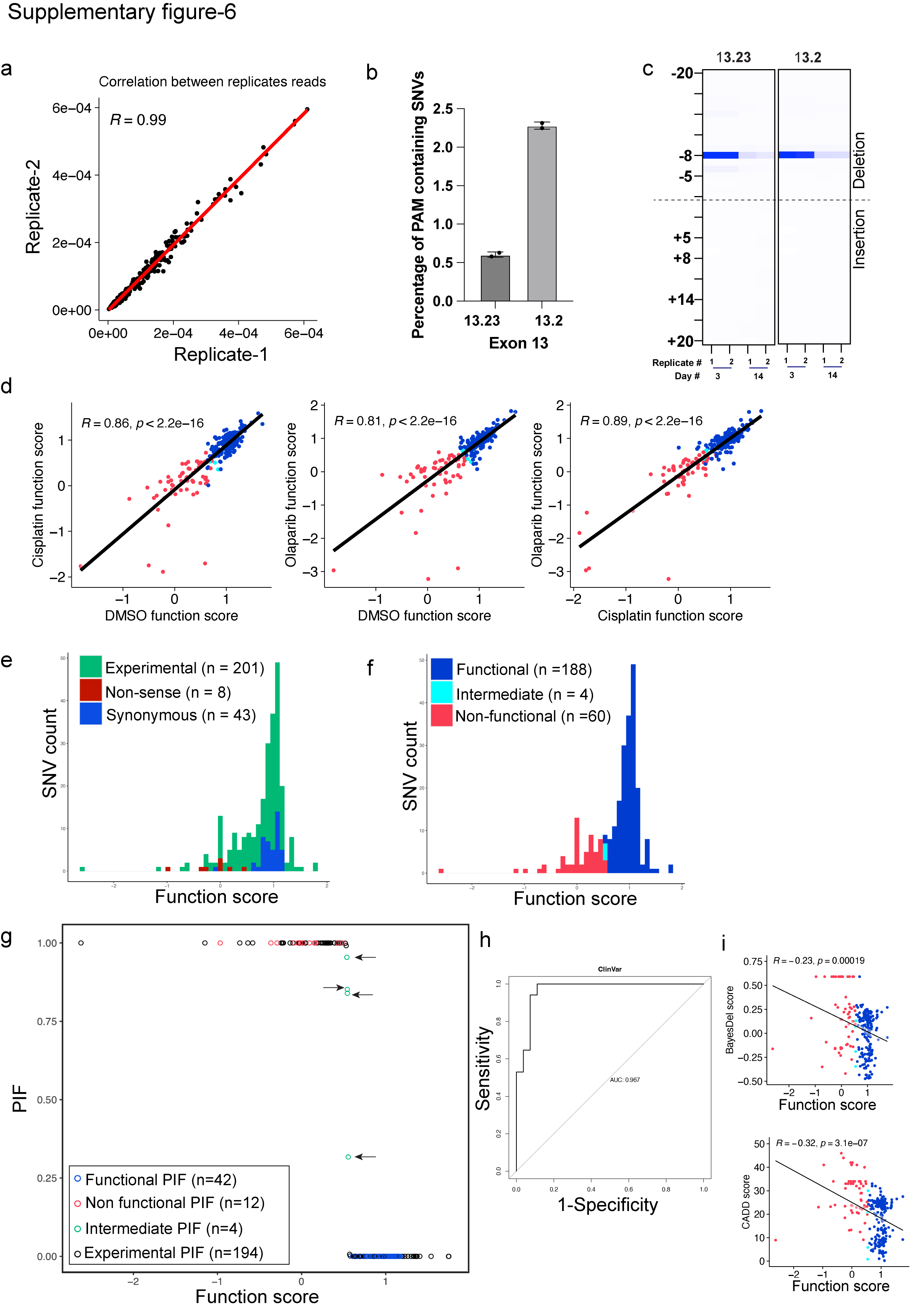

Supplement: S6 Fig — (a) Pearson correlation showing the read abundance for each variant at day 3 between two independent replicates of Exon 13, ρ = 0.99 for 252 SNVs of Exon 13. (b) Percentage of HDR as quantified by the proportion of SNVs containing the PAM modifications. (c) Heatmap showing the distribution of indels formed at each exon. The frequency is normalized to the total read counts and z-score for each base pair indels is plotted. Each lane corresponds to the two independent replicates. (d) A strong correlation of the function scores between two independent replicates for 252 SNVs for DMSO vs cisplatin (R = 0.86), DMSO vs olaparib (R = 0.81) and cisplatin vs olaparib (R = 0.89). Red dots represent nonfunctional, blue dot represents functional and gray dot represents intermediate category of variants. (e) Histogram showing the distribution of function scores of 252 SNVs of which 8 SNVs are known to be non-functional as it encodes for a stop codon, 43 variants are known to be functional as it does not change the amino acid. Remaining 201 variants were experimental and their function scores were bimodally distributed. (f) Histogram showing the categorization of 252 variants into functional (188 SNvs), intermediate (4 SNVs) and non-functional (60 SNVs). (g) The distribution of PIF calculated based on the distribution of function scores revealed a clear delineation between functional and non-functional SNVs and only 4 variants were in the intermediate zone. (h) ROC plot showing the sensitivity and specificity of classifying the ClinVar-reported variants from exon 13. (AUC = 0.967) (i) Negative correlation between SGE-derived function scores of 252 SNVs with Bayes-del score (ρ = -0.23) and CADD scores (ρ = -0.32). Blue dots represent the functional, cyan dots are intermediate and red dots are non-functional variants according to our PIF-based calculation. (TIF) [file pgen.1010940.s006.tif]
