# Supplementary material for: Saturation genome editing of 11 codons and exon 13 of BRCA2 coupled with chemotherapeutic drug response accurately determines pathogenicity of variants
Source: PLoS Genet. 2023 Sep 15;19(9):e1010940. doi: 10.1371/journal.pgen.1010940 (PMC10529611; doi:10.1371/journal.pgen.1010940)
Supplement: Attachment — Submitted filename: Sahu et al_Response to reviewers_PGEN.docx [file pgen.1010940.s011.docx]

**Response to reviewers’ comments (PGENETICS-D-23-00242)**

**Saturation genome editing of 11 codons and exon 13 of *BRCA2* coupled with chemotherapeutic drug response accurately determines pathogenicity of variants**

We would like to thank all the reviewers for their constructive comments and valuable suggestions. These have helped us to significantly improve the overall quality of the manuscript. We have addressed all the concerns of the reviewers and have made necessary changes in the manuscript.

Specific changes made to individual figures to address the reviewer’s comments are as follows:

1. **Figure 1:** Performed IR-induced RAD51 foci formation and EdU pulse labelling experiments and added new data in Fig. 1f and g showing the functional equivalence of single copy BRCA2 expressing mouse ES cells with parental mESC clone.
2. **Figure 2:** Represented the distribution of function scores for non-sense and synonymous variants in a swarm plot.
3. **Figure 3:** Revised the sequence-function map for enhanced visualization for all the single nucleotide variants across 11 codons (fig.3a), Added new data on no irradiation control for all the BRCA2 variants used for targeted validation (fig.3c).
4. **Figure 4:** To address the scalability of our saturation genome editing approach, we have added new data for all possible single nucleotide variants of exon 13.
5. **Supplementary figure-2:** Added correlation plots for read counts separated by individual exon (fig. S2b), Indel formation and HDR Editing efficiency for individual exons across 11 codons (fig. S2e).
6. **Supplementary figure-4:** Formatting of the plots to represent all data points under same axis (Fig.S4 a-d)
7. **Supplementary figure-5:** Added correlation between functions cores and CADD and BayesDel scores for all SNVs (fig S5 b-c), Heatmap showing the distribution of PIFs for variants suggesting the impact of DNA damaging drug sensitivity assay for variant categorization (fig S5d)
8. **Supplementary figure-6:** New data on exon 13 SGE experiments showing the QC, editing efficiency, distribution of function scores, correlation of PIF and function scores, correlation with CADD and Bayes Del score.

Our point-by-point response to the reviewers’ comments is described below:

**Reviewer #1:** In this paper the authors perform saturation genome editing of a region of BRCA2, an important breast and ovarian cancer gene. The manuscript presents a novel humanised-mouse ES cell model which is functionally validated - the text and figures are generally clear. I have several specific comments on this paper.

We thank the reviewer for their positive assessment of our manuscript. Their recognition of our efforts and the innovative use of humanized-mouse ES cell-based assay for functional classification is truly encouraging. Please see below our response to specific comments.

1. Throughout the manuscript the authors call VUS “variants of unknown significance”. ACMG calls VUS, variants of uncertain significance.

We thank the reviewer for pointing this inadvertent mistake and we have revised the manuscript calling VUS as “Variants of uncertain significance”.

In a similar vein the authors state that “Large-scale genetic sequencing has not been widely used as a clinical paradigm due to our limited ability to determine the functional impact of genetic variants. “ – I would argue this is far from the situation with genetic testing programmes expanding exponentially.

We concur with the reviewer’s observation that genetic testing has become extensively utilized. However, as the scope of sampling and genetic testing expands, a greater number of distinct missense variants are being identified. Consequently, this has resulted in the identification of numerous VUSs, emphasizing the critical need to ascertain the clinical significance of these VUSs. We have revised this sentence to incorporate the reviewer’s suggestion. (Page 3, authors summary, 2^nd^ sentence).

I also think that the use of "functional" and "non-functional" variant in the paper might confuse some people. Maybe "disruptive" and "non-disruptive" is better.

We have followed the standards and guidelines mentioned by ACMG in Richards et al., 2015 and categorized our variants into “functional” or “non-functional”.

2. The paper describes editing of just 11 codons. I was unable to find a clear description of why the authors selected these 11 codons and feel this is important for a general genetics journals such as Plos Genetics**.**

We regret that we did not provide the rationale behind the selection of specific codons in this study. We have provided this information in the revised manuscript on page 6 (2^nd^ paragraph). The selection of these residues was based on several factors. Firstly, certain residue, such as p.T2722, p.D2723, and others mentioned in fig. 3d, have been extensively investigated by numerous laboratories. This widespread study of these residues allowed us to utilize them as established controls, distinguishing between functional or non-functional, thereby serving as reference points for our assay. Secondly, variants were randomly selected across few exons to ensure the applicability of our approach across various regions of the gene. This selection allowed us to investigate the functional relevance of key domains in BRCA2. Interestingly, our findings indicated that alterations in residues at p.D2723 in exon 18 and p.W2788 in exon 19 resulted in a higher proportion of non-functional variants. In contrast, variants corresponding to residues at p.Y57, p.E58, p.P59 in exon 3 exhibited an overall benign nature (as shown in Fig. 2d). Furthermore, sgRNAs were selected based on SpliceAI predictions such that the synonymous PAM mutation does not affect canonical splicing.

Of note not all possible changes were analysed and there is some missing data making this a relatively small dataset. Only one gRNA was used.

In this study, we have made efforts to target all possible combination of variants using this approach. However, it is important to note that a few variants were excluded due to low frequency of reads, which can be attributed to limited sequencing depth. The inclusion of low-read count variants can impact the accuracy of classification of these variants. In summary, each codon position can give rise to total 63 non-wild type variants. Out of 693 possible combinations of variants corresponding to residues at p.Y57, E58 and P59 of Exon 3; T2722, D2723, G2724, C2765, P2767 and L2768 of Exon 18; T2783 and W2788 of Exon 19, we have successfully generated and classified 599 possible combinations of variants, accounting for 86.4% coverage. (Page-9, 2^nd^ paragraph)

While employing a single gRNA is generally considered a preferable approach due to the ability to make minimal PAM synonymous substitutions, it does impose limitations on saturating large fragments. To overcome this, we have included new data for exon 13. In this case, we have incorporated all possible single nucleotide changes using three distinct sgRNAs. We have classified 95.4% of total possible SNVs in exon 13. Please see page 18-21, Fig 4, Supp. fig.6.

3. In looking at Figure 2/3 it is clear that there is not a big delineation between "functional" and "non-functional" (disruptive and non-disruptive) variants. To what extent does this influence the utility of these method for analysis of the whole gene and indeed have the authors considered using different models to try and resolve more of the intermediate variants?

We appreciate the reviewer’s insightful suggestion, which has enabled us to provide additional information and strengthen the robustness of our study. In our study, we employed three functional assays to assess the variants: DMSO as a readout for normal cell growth; and cisplatin and olaparib as readouts for sensitivity to DNA damaging agents. This comprehensive approach allowed us to effectively categorize the majority of variants as either functional or non-functional with high specificity and sensitivity. Fig 2e clearly illustrates a distinct separation between functional and non-functional variants, with no overlap observed between these classes. Only a small subset of variants (8 in total) fell into the intermediate category.

The significance of this method extends beyond the specific variants investigated in our study. With the success we have achieved, this tool can be expanded to classify all possible missense variants of BRCA2. In response to the reviewer’s suggestion, we have included new data in the revised manuscript that demonstrates the classification of all possible single nucleotide variants for exon 13 of *BRCA2.* (Please see fig. 4).

In sum this is an interesting model system for exploring BRCA2 albeit described here with a very small dataset.

We thank the reviewer for appreciating our work and providing thoughtful and constructive suggestions to enhance the quality of our manuscript.

**Reviewer #2:** Sahu and colleagues report a pilot-scale saturation genome editing scan of the tumor suppressor gene BRCA2. They develop a mESC model bearing a single copy of human BRCA2 on a BAC transgene, which they then subject to multiplex CRISPR/Cas9-medaited editing to knock in “NNN” codon libraries across 11 selected codons. Deep sequencing is used to measure the resulting allelic fractions at two timepoints as well as after drug treatment, which then contribute to a score taken as an estimate of the likelihood of pathogenicity. BRCA2 contributes a heavy load of clinical VUS, so scalable approaches to generate function data in its variants are needed. This manuscript takes a modest step in that direction, though it leaves out important details and analyses as I describe below.

We thank the reviewer for recognizing the potential of our assay. We appreciate the suggestions and concerns. We have taken these suggestions into consideration and have addressed all the concerns as described below.

Major issues
- Scale. The experimental system shows promise as a platform for BRCA2 VUS functional profiling. However, targeting only 11 codons does leave open the question of whether it will be feasible to scale to all >3000 codons cDNA or to the undefined number of codons which contribute to the VUS burden.

The significance of this method extends beyond the specific variants investigated in our study. With the success we have achieved, this tool can be expanded to classify all possible single-nucleotide variants (SNVs) of BRCA2. In response to the concern regarding scalability, we have included new data in the revised manuscript that demonstrates the classification of all possible single nucleotide variants for exon 13 of BRCA2. (Please see fig.4 and supp. fig. 6). Through this expanded analysis, we have successfully classified 252 SNVs out of 264 possible SNVs, accounting for 95.4% coverage, with a 95.45% specificity and 100% sensitivity. This additional data demonstrates that our assay has the capability to be scaled up for the saturation of all codons of BRCA2 in future studies. We appreciate the reviewer’s feedback, which has enabled us to reinforce the potential of our assay for broader applications. Please see page 18-21, Fig 4 and Supp. fig.6.

- Drug selections. Function scores from the drug treatments (cisplatin, olaparib) are very closely correlated with function scores from outgrowth in DMSO (comparably so to DMSO-DMSO replicate correlations). It was not clear whether these selections added any information beyond that of additional replicates. Did any individual alleles behave qualitatively differently under either/both of those selections vs in DMSO?

We agree with the reviewer that the drug treatments are closely corelated in our dataset and as suggested we have identified variants that have a PIF score <0.5 (nearly functional) and we observed that 33 variants out of 599 variants are sensitive to either or both cisplatin and olaparib treatment. This suggests that the incorporation of cisplatin and olaparib to the experimental approach, in addition to cell growth allowed us to critically score the functional impact of these variants. We have added this data in revised supp. fig. 5d.

- Sequencing data processing.
o Basic QC characteristics of the sequencing data were not shown. E.g., what fraction editing was obtained? What level of background mutations arise purely from sequencing error (sequencing WT cells or no-Cas9 transfection)?

We have calculated the percentage of indels formed per experiment using each sgRNA and they generally range from 17 to 40% and the percentage of HDR ranges from 0.3 to 1.5 %. Please see Supp. fig. 2e.

We observed the background mutations generally occur at a frequency less than 1 in 10^5^ reads. Moreover, it is extremely rare to get the PAM mutation at the specific site and additional two or three nucleotide change at the position where we expect our mutations to be present. This read counts threshold is also based on previous BRCA1 saturation genome editing performed by Findlay et al., *Nature,* 2018.

o Different exons’ function scores (Fig S2b) and editing frequencies (Fig S2c) seem to span very different ranges. Are there any variants (e.g., synonymous SNVs) that could be used to normalize the exons to one another?

We thank the reviewer for bringing this to our attention, which we inadvertently missed to explain in the original manuscript. The variability in editing as well the position of the nucleotide from the sgRNA cut site may influence variant recovery. This was not prominent in NNN degenerate oligo-based experiments as all possible combinations were across 3bp of the codon and the residues were at proximity of the cut site. However, this was important when we saturated the entire exon 13 to generate all possible single-nucleotide changes. Since exon 13 saturation was performed in two parts, the scores were normalized such that the two experiments can be modelled together. The position biases in editing rates were modelled using day 14 over day 3 ratios and chromosomal position for each SNV and within each experiment. The “loess” function from R was used to fit the log2 of this ratio as a function of chromosomal position. Only the SNVs with a ratio 1.25 were used to limit influence of biological effect in the model. The model thus provided a score for each chromosomal position corresponding to position biases.  For each SNV, this score was subtracted from the log2 of the day 14 over day 3 ratio.

The corrected log2 day 14 over day 3 ratios were linearly scaled within the experiment. Then, the obtained function score was normalized across the experiments such that the median synonymous and nonsense function scores were the same as the global scores across the exon.  A gaussian mixture model was used to estimate the probability for each SNV to be pathogenic or benign, using the function score. We have provided a detailed explanation on this strategy in the revised manuscript. Please see Material and methods section #9 on SGE of all possible SNVs from exon 13 (Page 28-29)

o Figure 2c. is there a single cutoff that perfectly separates the nonsense and synonymous variants? This is a small enough number of points (n=21 and 29) that they should be shown as individual measurements (e.g., in a swarm plot) instead of using binning, which obscures details.

We thank the reviewer for the suggestion and have now plotted the data as a swarm plot. Please see revised fig. 2C which shows clear delineation in the function scores between nonsense and synonymous variants.

- Exon 3 effects. As seen in Fig 2b and S2d, frameshift indels in exon 3 seem to persist without fitness defect between day 3 and 14. What might explain this apparently prevalent false negative? Does this affect interpretation of other effects (or lack of) in that exon?

We acknowledge the reviewer’s comment regarding the persistence of variants with frameshift indels. While we do not precisely understand how variants with a single nucleotide deletion are able to persist, it is worth noting that the majority of out-of-frame indels exhibit a significant fitness impact. Additionally, there is a decrease in the occurrence of stop codons from day 3 to day 14, and synonymous variants are present in the variant pool. These observations suggest that the functional interpretation of the variants in this exon remains largely unaffected.

- Validation experiments.
o Why are these particular missense variants chosen (beyond being MNVs and therefore not previously well studied)?

The missense variants were randomly chosen to validate our high throughput NGS results. Our intention was to investigate whether multi-nucleotide variants that exhibited functionality in our assay would behave similarly when targeted mutations were introduced using CRISPR-Cas9.

However, we want to clarify that the non-functional variants were not generated in the *Brca2^-/-^ BRCA2 (Tg)* ES cell line. This is because a loss-of-function variant is incapable of complementing the lethality resulting from the loss of *Brca2.* Therefore, we focused primarily on functional variants, as we aimed to assess their functional impact.

o In Fig 4A, WT curves seem different between the bottom and top – do these represent different experiments?

We acknowledge the variability observed between different experiments and the minor batch-to-batch variations in the drug sensitivity assays. To address this, we implemented a rigorous control strategy in our experimental design. In each batch of experiments, we included a wildtype (WT) control as a reference point for all our analyses. By including a WT control in each batch, we were able to account for any experimental variability or batch-specific effects. This approach allowed us to normalize and compare the results within the context of the specific experimental conditions, ensuring the accuracy and reliability of our findings.

No synonymous control is shown for exon 18, and p.Leu2768Lys is mentioned in the text but does not appear in the figure

We have used a wildtype control in this experiment. We apologize for the inadvertent typo in the text. We did not validate p.Leu2768Lys variant. We have now deleted p.Leu2768Lys from the text (page 17)

- Splicing effects are mentioned several times but there are no results nor analyses to support these. How sure can we be that the up to 3 bp changes introduced, plus the PAM-ablating mutation, do not affect splicing? A though comparison should be made between any previous evidence for/against splicing disruption and the measurements made here. In addition, SpliceAI or similar should be used to score the sequence level changes made here to see how many of these effects could be attributed to splicing.

We thank the reviewer for this important clarification, and we have incorporated these details in the revised manuscript. Recognizing that PAM-ablating mutations have the potential to impact splicing, we utilized tools such as SpliceAI. This was one of the rationales behind selecting specific sgRNAs and residues in our study. We have explained this in the revised manuscript (page-9, 2^nd^ paragraph). To address the concern regarding splicing, we have included additional results from SpliceAI in the revised manuscript. These results indicate that the synonymous PAM modifications we used have no apparent effect on splicing. We do not see any significant changes in donor gain, donor loss, acceptor gain and acceptor loss based on SpliceAI scores.

- Comparison to other high-throughput functional assays. How well correlated are these results to those of Ikegami (ref 21), Hanna et al (ref 27), and Cuella-Martin et al (ref 28)?

We have compared our functional classification to other high throughput assay data like Ikegami et al. Please see revised figure 3A that summarizes the variants that overlap with Richardson et al., 2021, and Ikegami et al. 2020.The base editing screen by Hanna et al., 2021, comprises of 268 variants, of which only Asp2723Asn and Thr2783Ile were common to our dataset. It was not feasible for us to compare our dataset with Cuella-Martin et al 2021 paper due to the lack of enough common variant.

- Written clarity. The text ends abruptly without discussion which could help place these results in the context of other functional studies or additional lines of evidence. The writing is at times hard to parse or unclear.

We appreciate the reviewer’s feedback regarding the written clarity of our manuscript. We have provided a clear and comprehensive discussion that contextualizes our results within the broader field of research and other lines of evidence. We have made the following revisions to address these concerns:

1. **Discussion of results in the context of other functional studies:** We have expanded the discussion section to include a thorough examination and comparison of our results with relevant studies in the field. This allows us to highlight the similarities, differences, providing a better understanding of how our work fits into the existing body of knowledge.
2. **Additional lines of evidence:** We have incorporated references and discussions related to additional lines of evidence that support and complement our findings. This includes referencing other studies, experimental approaches that further strengthen the validity and significance of our results.
3. **Improved writing clarity**: We have carefully reviewed the manuscript to enhance the overall clarity and readability of the text. We have revised and rephrased sentences where necessary to ensure that the information is conveyed concisely and is easy to comprehend. Additionally, we have paid extra attention to the logical flow of ideas, to ensure that each paragraph and section contributes coherently to the overall narrative.

- Variant nomenclature. Protein level variants are referred to extensively in the text, which made it unwieldy to connect these to cDNA-numbered variants e.g., shown in Figure 3e. The text should mention the “c.” numbers for each variant as well as protein level change.

We thank the reviewer for the suggestion and have now revised the manuscript with cDNA-numbered variants in the main text.

Minor
- Fig 1b, what is being probed?

We apologize for missing out this critical information and have now revised it in the figure legend. Probes targeting the mouse *Brca2* (conditional and knockout allele) and the human *BRCA2* was used in the Southern blot.

- In 1c, what is the expected copy number and what conclusions are drawn from the Tg measurements being > those of TK?

To ensure the accurate integration of the transgene, we employed various methods and tools, as outlined in Figure 1 of our manuscript.

One of the key validation steps involved the use of the *Thymidine Kinase (Tk)* gene. A single copy of *Tk* was targeted to the *Brca2* locus as described by *Kuznetsov et al,* 2008. By assessing the intensity of the band corresponding to *Tk* on a Southern blot, we were able to confirm the single copy integration of the *BRCA2 BAC Tg*. This validation was crucial in establishing the integrity of the transgene integration.

Additionally, we utilized a combination of other robust techniques to further validate the single copy integration. These included western blot analysis, and chromosome FISH. These complementary approaches collectively supported and reinforced the evidence of single copy integration of the *BRCA2 BAC Tg* construct. We also performed a series of genome editing experiments, where we have seen that out-of-frame indels led to cell lethality, further confirming the single copy integration.

- is the antibody specific to human BRCA2 such that this blot shows only the human gene’s expression? If the case, this does not demonstrate equivalent expression between the transgene and endogenous copies.

Yes, the antibody is specific to human BRCA2 with no cross reactivity to mouse Brca2. This antibody has been validated in numerous studies including Sullivan et al., *Human mutation* 2021, Biswas et al., *Blood,* 2011.

- Fig 3a. PIF scores as previously shown are probabilities. In 3/3b, they are shown ranging from -1 to 2. Specify how they were transformed.

We apologize for the error. We have revised these plots as correlation between function scores in cisplatin and olaparib and categorized by different PRIOR classes. Please see revised supp. fig. 5a.

- Fig 3. Why the switch from PIFs to single-assay log2 scores?

Fig.3 demonstrates the distribution of function score (Log2 scores) to show how CADD scores or Bayes Del scores are inversely proportional. This data suggests our PIF classified nonfunctional variants have a high CADD or Bayes Del score.

**Reviewer #3:** Specific comments

The title is misleading – implies a large-scale MAVE style study – the study is impressive compared to previous BRCA2 functional assays but at the same time covers a limited number of codons (11) in only 3 exons. The title should be adapted to justify why analysis of just the 11 codons selected was considered most relevant – this is not in the abstract either. There is mention of why much later in the m/s on page 11 but there is no indication of what proportion of the functional domain/s of likely clinical importance have been targeted.

We thank the reviewer for their thoughtful suggestions and finding our work to be “impressive compared to previous functional assays” and their suggestion to revise the title. We agree that this is a relatively small dataset. However, it serves as a crucial foundation for demonstrating the feasibility and potential of CRISPR-Cas9 based SGE tools to generate all possible variants of BRCA2 in the future. As suggested by the reviewers we have revised the title to highlight the specific focus on the saturation genome editing of 11 codons and exon 13 of *BRCA2* in this study.

Furthermore, in the abstract and on page-6, we have provided additional details regarding the rationale for selecting these 11 codons for our proof-of-principle study. We also emphasize how this approach can be scaled up in the future to cover the entire BRCA2 genes. In response to the concern regarding scalability, we have now included new data in the revised manuscript that demonstrates the classification of all possible single nucleotide variants for exon 13 of BRCA2. (Please see fig. 4). Specifically, we selected exon 13 as few variants in this region have been previously studied by several laboratories and biochemical characterizations have been performed. Moreover, this exon is outside of the C-terminal DNA binding domain and few pathogenic variants have been identified. Through this expanded analysis, we have successfully classified 252 SNVs out of 264 possible SNVs, accounting for 95.4% coverage, with a 95.45% specificity and 100% sensitivity. This additional data demonstrates the capability of our assay to be scaled up for the saturation of all codons within the BRCA2 in future studies. We appreciate the reviewer’s feedback which has enabled us to reinforce the potential of our assay for broader applications. Please see page 18-21, Fig 4 and Supp. fig.6.

- Very first line of abstract is poorly phrased, should be reordered.

We have rephrased this sentence.
- It is unclear what the BRCA consortium in ref 20 refers to

Ref 20 refers to a variant pathogenicity prediction from evolutionary data.

- It is unclear why references 35-38 are referred to as clinical repositories – they are previously published functional assay studies?

We apologize for the error. We have now referred to them as functional studies.

- Assuming by the language that the southern blot was to BRCA2, not sure where in the text the detail for what the southern blot probe design is, or what it detects?

We apologize for missing out this critical information and have now revised it in the figure legend. Probes targeting the mouse *Brca2* (conditional and knockout allele) and the human *BRCA2* was used in the Southern blot. The details on probe design is described in Kuznetsov et al., *Nature Medicine*, 2008 and we have referenced this work (Ref #36).

- Figure legend 1. “Neomycin resistant clones were o screened for the presence of BRCA2.” Unclear from the wording of this if neomycin was used to select clones, and validated for BRCA1 or if the mESC electoporated ells were selected by neomycin and then BRCA2 presence validated, if so, how was this validated? Please clarify this?

Neomycin was used to select mESC for the presence of BAC containing full length wildtype *BRCA2.* We then we confirmed the expression of BRCA2 by qPCR followed by western blotting. We have added this information in the revised manuscript (Please see section 2 in Materials and Methods.).

o What is F7 and F11, it is not in the legend, it is in the text but use consistency for clarity.

We apologize for not clearly explaining what F7 and F11 represent. We have now provided this information in the figure legend.

- **Figure 2**
o Fig 2c What is the possible explanation for the bimodal distribution in scores for stop codon variants?

The function scores of all the variants taken together in Fig.2C are bimodally distributed which means some are functional, and some are non-functional. The synonymous or the Stop codon variants do not show any bimodal distribution.

o It is stated that the synonymous variants have a positive score - perhaps there is misrepresentation of the numbering on the x axis, but it appears from the figure as if at least some of the synonymous variants have a negative score?

The function scores for synonymous variants ranged between -1 to +0.6 and we have mentioned this in the revised manuscript (page 10)

o 2d sloppy terminology – the data represented should be referred to as a measure of function not reflective of pathogenicity. There is no clinical classification represented that subfigure.

We agree with the reviewer that the heatmap represents the distribution of function score and not pathogenicity. We have rephrased the figure legend in the revised manuscript.

o 2e. Why are there no red or blue CI lines, and these are not referenced in the figure legend to clarify, assuming the three colours are the ‘vertical lines’? More explanation is needed to describe the various categories experimental etc etc. The term classification should not be used in this context, to avoid confusion with the final clinical classification (eg ACMG) for a given variant. Suggest categorization perhaps. The comment is made in the legend “Cis were of considerable size due to the model’s ability to perform sharp separation”. Can the authors explain this statement - CI for individual variants is a measure of confidence in the repeatability of results for a variant, surely. So do these results indicate that some variant repeats for the assay had readout of no function and others as functional??

For most of the variants, the confidence intervals (Cis) are negligibly small; that is why there are no visible blue CI lines (for functional controls) or red CI lines (for non-functional controls). Most of the black circles corresponding to the experimental variants are positioned so close that they form a continuous thick black line. We have added this information in the legend of fig. 2e. As suggested by the reviewer, we have changed the word “classification” to “categorization” throughout the manuscript.

The occurrence of a few large CIs could be expected and, during our statistical-model development, was associated with our model’s ability to perform sharp separation between functional and non-functional variants with the stringent PIF thresholds. For a few variants, this resulted in an increased sensitivity of the corresponding PIFs to the training-data randomization used in CI generation. We have explained this in the figure legend.

o 2f legend – again, please avoid the term classification in this context to avoid confusion.

We have revised the text and used as “categorization” instead of “classification” throughout the manuscript.

Pg 12 : the authors state “The results obtained from functional assays are not always binary due to the cell-line-specific differences, technical or experimental variability. Hence, the development of a statistical classifier to generate a function-impairment probability, such as PIF is useful for the integration of results from multiple functional assays, providing a more reliable assessment of the impact of VUS.” Not at all sure how relevant this is to this study since all assays were done in one line – can the authors explain what they mean? Is this actually referring to the three exposures used in the experimental design.

We meant the DMSO control as a readout to check for cell growth and the response to cisplatin and olaparib as a readout for the sensitivity of variants sensitivity to DNA damaging agents. We used function scores from the three assays to generate the probability of impact on function (PIF).

The comparison to bioinformatics methods is not very helpful. Bioinformatic prediction of protein impact is not reliable (which is why unless calibrated it is recommended to be used at only supporting evidence). As the authors state, similar comparisons have shown up discrepancies, so what value to do again?

We agree with the reviewer’s suggestion and the computational predictors are mostly taken as supporting evidence. We have removed this plot from the main figure and included it as supplementary data (please see supp. fig. S5) to support our functional categorization of variants.

A more meaningful comparison would be to previous functional assays for the subset of variants with such data available and/or comparison to classification assertions in clinvar (which they do to some extent later- a more comprehensive summary tabulated as supplementary information would be helpful and save a lot of text).

We agree with the reviewer and have revised the manuscript to include a comprehensive discussion of our functional categorization of variants and how it aligns with the findings of previously reported functional assay and ClinVar predictions.

We have included supplementary file-2 that provide detailed information on the variants, their functional categorization, and their comparison with previous studies and ClinVar. This will enable readers to easily assess and contextualize our findings within the broader landscape of variant classification in BRCA2.

“Furthermore, our variant classification is consistent with other functional assays” – again the term classification should not be used for categorization based on functional assay results alone.

We apologize for the mistake and have now revised the entire manuscript mentioning them as “functional categorization”

Figure 3 - the key has grey dots as intermediate but there are none in the figure.

We apologize for the error in the legend. There are no intermediate SNVs and we have now removed them.

Figure 3 a,b why are both needed? And where does the terminology for null etc come from

Based on the reviewer’s suggestion we have now removed these plots from the main figure and kept data from CADD, Bayes-Del and PRIOR. The terminology of “null”, “moderate”, “low”, “high”, “extreme” comes from the PRIOR class generated by the Huntsman Cancer Institute. (<http://priors.hci.utah.edu/PRIORS>).

“p.Pro2767Ser is identified in a patient with a benign cancer.” No cancer type or reference is provided.

The p.Pro2767Ser (c.8299C>T) BRCA2 (rs587782619) variant was identified *BRCA1/2* NGS screening in a young woman who was 26 years old when diagnosed with breast fibrocystic dysplasia. This is considered to be a benign condition. This variant was confirmed on the patient’s DNA by standard Sanger sequencing and was not found in any of the remaining 849 subjects that were examined. The allele frequency of this variant was 5.8 × 10^−4^. Molecular testing did not identify any known pathogenic mutation in the *BRCA1* or *BRCA2* genes. The BRCA2 p.Pro2767Ser was annotated on the ClinVar database as a VUS (Esposito et al., Cancers 2019). We have now provided this information in the revised manuscript (page 15).

“Exon 3 is a critical domain for PALB2 binding and has been previously reported to include several variants affecting splicing (51).” It is not clear what relevance this statement has to the discussion at that point which appears to be around missense variation?

We agree with the reviewer and have now deleted this sentence.

“Since BRCA2 variants that are non-functional and will not survive in Brca2ko/ko mESCs, we have focused on the variants that are functional based on our PIF classification” - remove the word “and”.

We have revised this sentence.

Please justify the rationale to designate p.Leu2510Pro as hypomorphic.

We first reported the functional characterization of p.Leu2510Pro in *Biswas et al,* *Blood* 2011, where the mESCs expressing this variant were reported to survive at a low frequency and were highly sensitive to DNA damaging agents. We recently reported a knock-in mouse model of this variant (*Mishra et al, Nature Communication, 2022*). We showed that while homozygous and hemizygous mutant mice were born alive, they were born in skewed Mendelian ratios. A significant fraction of mutant mice died during embryogenesis. Embryonic and adult fibroblasts exhibited impaired loading of IR-induced RAD51 foci and were sensitive to DNA damaging agents. Both these papers support the hypomorphic nature of p.Leu2510Pro variant and have been referenced in the revised manuscript (Page 17, references #37 and #56).

- Supp Figure 4d – explain what experimental means – assumedly the variants that are not truncating or synonymous? The intermediate variants are supposed to be in grey –not clear where they are in the figure. Can they be marked with a hash or something? Do the figures on the RHS include controls – apparently since the n=599 is the same as on the LHS. - Please highlight the intermediate variants reported with PIF values in the figure – hard to place them in context with the other variants.

The experimental variants are the variants that are not truncating (stop codons/non-functional) or variants that do not change the amino acid (synonymous/functional). The intermediate variants (n=8 out of 599 variants) which is a very small number hence they got embedded between the red (Pathogenic) and blue (neutral) bars. As suggested, we have used yellow asterisk to identify the intermediates.

- **Figure 4**
o Why are the control cells at a different magnification to the variant expressing cells. Do you have an IR negative or a

We have revised this figure with cells at same magnification. As suggested by the reviewer, we have performed RAD51 foci quantification without any ionizing radiation and have now presented the data in Fig. 3C.

- Methods section 7 – change functional classification to categorization – again to avoid confusion with ACMG terminology.

We have revised and changed the term to “categorization” throughout the manuscript.

General comments
- The methods are throughout the main manuscript, methods and figure legends, and it would appear that details of the methods are missing, though it is hard to be sure with the way it has been written.

We have extensively revised the results and discussion section with key details described in the methods section.

- The selection of 1 single clone to represent a humanised mESC line is questionable. Would like the authors to comment on the similarity or difference in behaviour of the single clone, relative to the parental line. Doubling time, behaviour etc.

We appreciate the reviewer’s suggestion regarding the concept of biological replicates in *in vitro* cell-culture based experiments. To address the concern about the similarity of the single ESC clone to the parental ESC line, we have performed additional experiments to provide evidence of functional similarity. Specifically, we have conducted the following experiments-

a) We have investigated the formation of IR-induced RAD51 foci, which is indicative of the ability of BRCA2 to recruit RAD51 to the sites of DNA double strand breaks. Our results, demonstrate that the selected mESC clone exhibits comparable levels of RAD51 foci when compared to the parental line. This suggests that the single clone retains the functional capacity of BRCA2 in loading RAD51 and is functionally similar to the parental ESC line in this aspect. (please see Figure 1f).
b) Additionally, we have conducted EdU pulse labeling experiments to assess the doubling time of the single clone. The results indicate that there is no defect in the ES cell doubling time, further supporting the functional similarity to the parental line (please see Figure 1g).

We have included the new findings in the revised manuscript and aim to provide evidence of the functional equivalence between the single clone and the parental ESC line (page 8, 1^st^ paragraph).

Training set of variant is “21 functional synonymous variants with no change in amino acid and 29 non-functional variants generating a stop codon”. Using loss of function variants as a predictor of missense variant pathogenicity might be questioned, given the different possible mechanisms by which a missense variant might impact the protein function. It would be helpful to have existing known pathogenic and benign variants designated, and used for a formal calculation of Likelihood ratio towards pathogenicity as recommended by Brnich et al.

We agree with the reviewer’s perspective and acknowledge that missense variants with no amino acid change can still have non-functional consequences due to nucleotide changes affecting splicing. To address this, we performed SGE using NNN degenerate oligos, which not only generated all the possible combinations of variants but also generated substantial number of synonymous and stop codon variants. These variants were included in our training dataset to evaluate their functional impact and to assess their classification using our assay.

To validate the reliability of our approach, we compared our functional classification results, with the ClinVar database, which contains reported variants with clinical significance. We observed a strong overlap between the classification provided by ClinVar and categorization based on our functional data, suggesting that our assay is consistent with the known classification of these variants. This alignment further supports the robustness of our functional categorization approach.

It is worth noting that this approach of including synonymous and stop codon variants in the training dataset to assess the performance of functional classification has been employed in previous large-scale SGE studies, such as the SGE of BRCA1 dataset (Findlay et al., *Nature* 2018).

The likelihood ratio calculation as described in Brnich et al., typically requires multiple lines of evidence, including clinical data. However, in our study, we didn’t have access to patient-specific clinical data for the variants analyzed. Instead, we calculated positive LR and negative LR to provide an indication of the probability of a variant being pathogenic based on our functional assay results. The positive LR represents the likelihood of a variant being truly pathogenic, while the negative LR represents the likelihood of a variant being incorrectly assigned as benign, when it is actually pathogenic. It is calculated by the following formula:

Positive LR = sensitivity / (100 – specificity). 
Negative LR = (100 – sensitivity) / specificity.

In our exon 13 SGE dataset, we determined the sensitivity and specificity of our statistical model and obtained LR values for the exon 13 SGE dataset. By reporting these LR values, we aim to provide insights into the probability of a variant being pathogenic based on our functional assay results. While a comprehensive LR calculation as described in Brnich et al., may require additional clinical data and multiple lines of evidence which is currently beyond the scope of this study. We believe that the LR+ and LR- values that we have provided offer useful information regarding the likelihood of a variant being pathogenic or benign within the context of our functional assay. We have now provided these results in the revised manuscript (Page-19) and in the methods section (Page-30).

- It’s unclear from any of the text if there are any biological replicates. “two independent replicates” were used, but these appeared to be performed concurrently. The biological variation in variant reads is not clear, the authors should clarify if the two independent replicates were performed in completely independent experiments and how biologically variant these were (though the use of the same clone as ‘independent’ is also questioned). This also might be supported by combining the figures in SUPP 2c, though duplicates does not really suffice for showing variability in an assay.

We appreciate the reviewer’s feedback and their concern regarding the terminology used to describe the replicates in our cell-culture based experiments. We have used the term “independent replicates” to describe the repetition of the SGE experiments. These correspond to two independent experiments performed using the same clonal mouse ES cell line, and the frequency of variant generation in these replicates is subject to stochastic variability. Even if a new frozen vial of cell stock was used, ultimately all cells in *in vitro* cell culture-based experiments originate from the same starting material, therefore achieving a true biological replicate is not feasible.

Majority of the previous large-scale saturation genome editing experiments relied on a clonal population of established cell line, like human haploid cell line HAP1 (Findlay et al., Nature 2014 and Nature 2018) and the haploidized HEK293T cells (Erwood et al., Nature Biotechnology 2020). Moreover, our PL2F7 mESC line has been used for functional classification of *BRCA2* variants since 2008 and has been validated and widely used by other laboratories as well. We also cross validate our functional data with other orthogonal assays developed using different cell lines and by different laboratories, suggesting clonal variation is minimal.

It is important to note that the clonal variation in transgene expression (BRCA2 in our case) could potentially lead to erroneous classification if different clones were used. By using a clonal population, we aimed to minimize such variation and ensure consistency in the functional scores calculated and correlated between the replicates. We have explained the concept of replicates in our assay in the revised manuscript in the material and methods section on “Nucleofection for CRISPR-Cas9 based saturation genome editing.

- Can the authors comment on the variability of reps for the variants called intermediate?

We have identified 8 intermediates out of 599 variants (~1.3 % of total variants) that we were unable to categorize. We would like to mention, that 4 of them can be called as “likely non-functional” as their PIFs tend toward non-functionality and 2 other variants tends towards functional nature. We found p.C2765L and p.L2768Q has a PIF in the intermediate zone.

| **Exon** | **Protein change** | **Codon change** | **PIF** | **Outcome** |
| --- | --- | --- | --- | --- |
| 3 | E58V | GAA>GTG | 0.28 | Likely functional |
| 18 | G2724S | GGG>AGC | 0.21 | Likely functional |
| 18 | C2765L | TGT>TTA | 0.43 | Indeterminate |
| 18 | P2767R | CCT>CGA | 0.97 | Likely non-functional |
| 18 | P2767A | CCT>GCG | 0.88 | Likely non-functional |
| 18 | L2768P | CTT>CCC | 0.10 | Likely functional |
| 18 | L2768S | CTT>AGT | 0.92 | Likely non-functional |
| 18 | L2768Q | CTT>CAG | 0.65 | Indeterminate |

The differences between the function scores of replicates is less than 2 which was a threshold used in Findlay et al., *Nature* 2018 paper, hence we think the variability did not influence the categorization.

Overall the paper is not easy to read and figures need more explanation in the figures themselves and the legends.

We have carefully considered this comment and have made significant improvements to enhance the clarity and comprehensibility of the manuscript. To address the concern about readability, we have revised the text to improve its flow and organization. We have focused on making the content more concise and ensuring that the key findings and conclusions are clearly presented. Additionally, we have paid close attention to the overall structure of the paper to enhance its readability.

We have revised and expanded the explanations provided in the figure themselves to provide a better understanding of the experimental set up and the results. We have also revised the figure legends to include more detailed descriptions of the data. We believe that these revisions, including the improvements made to the text and the figures, have significantly improved the clarity and readability of the manuscript.

Formal comparison to previous larger-scale studies is essential as is calculation of LR towards pathogenicity using ACMG recommendations – particularly since the authors stress many times how important functional information is for classification.

We thank the reviewer for raising this important point and agree that it is essential to have a comprehensive analysis of the functional information and its impact on variant classification. In our revised manuscript, we have expanded the discussion to include a comparison of our findings with previous larger scale studies that have investigated functional effects of BRCA2 missense variants. We correlated the functional consequences which provides valuable insights into the consistency of our results with existing literature.

We determined the sensitivity and specificity of our statistical model and used these values to calculate the positive LR and negative LR for the variants from the exon 13 SGE dataset. By reporting these LR values, we aim to provide insights into the probability of a variant being pathogenic based on our functional assay results. While a comprehensive LR calculation as described in Brnich et al., may require additional clinical data and multiple lines of evidence which is currently beyond the scope of this study. We believe that the LR+ and LR- values that we have provided offer useful information regarding the likelihood of a variant being pathogenic or benign within the context of our functional assay. We have now added these information in the manuscript (page 19).

It would be helpful also for the authors to state explicitly if/how the assay captures potential effects on splicing and which variants in their assay might be considered to be deleterious due to this mechanism of impact.

We agree with the reviewer that identifying variants that could be non-functional due to potential effects on splicing and this information will enhance the clarity and comprehensiveness of our research findings. This will also provide the assay’s scope to detect splicing-related effects, enabling a more thorough interpretation of the results.

In our current assay, the selection of sgRNAs for SGE was based on careful consideration of spliceAI predictions to ensure that PAM substitutions do not impact splicing. Ongoing studies in our laboratory have implicated cases where these PAM substitutions at the splice site significantly impact variant classification due to their effects on splicing. Hence, we believe that our humanized-mESC-based assay holds great potential for investigating splicing defects, and as we continue to saturate the entire BRCA2 gene, we anticipate identifying several variants with splicing implications. In response to reviewer’s suggestion, we have now included the results of our SpliceAI predictions in the revised manuscript, evaluating the effects of SNVs across all possible combinations in the 11 codons. Please see supplementary file-4.

**Reviewer-4**

BRCA2 MAVE Review
This is a high throughput study of BRCA2 function aimed at providing a line of evidence towards variant interpretation. The functional study uses a mouse embryonic stem cell line effectively null for mouse Brca2 and restored with a single copy of human BRCA2 (from a BAC clone-introns intact) harboring saturation mutagenesis covering nearly every single- double- and triple-nucleotide variation as well as NHEJ-driven indels targeted to select codons within three exons of BRCA2 using CRISPR/CAS9 technology. The cells are further exposed to a platinum and a PARP inhibitor and the pool of cells repleted or depleted are measured and transformed into a measure of cell survival and the functional score from each drug assay was synthesized into a statistical model for functional categorization as functional, non-functional and intermediate.
Similar methods have been employed effectively and at-scale in the recent past for other cancer predisposition genes and correlated well with clinical variant classification (e.g. for BRCA1, MSH2, etc.) and have been used successfully to further classify variants of uncertain significance. This is a novel approach for BRCA2 and although quite limited in scope at this time, will be a welcome addition to the arsenal of evidence used to classify variants in BRCA2 which are otherwise plagued by limitations around causing a relatively common phenotype: breast cancer.

Although this paper is relatively well written and mostly clear, I would like to suggest the following to further improve this manuscript:

We thank the reviewer for their positive assessment of our manuscript and for finding our approach to be novel with potential to utilize it to classify variants in BRCA2. We agree that the current limitation exists in the classification of BRCA2 variants, particularly due to the relatively common phenotype of breast cancer associated with these variants. We are pleased that our study can contribute to addressing these limitations and provide valuable evidence for variant classification. We have addressed the remaining concerns and incorporated the suggestions for data visualization as described below.

1.      The individual variant and its respective scores should be provided in a supplementary file in accordance with the guidelines put forward by the Mutational Scanning Working Group (PMID 31862013, recommendation 3)-I see the intention is to deposit them to a database upon publication, but I would urge this to also be a supplementary file tied to the publication.

We have provided the individual variant and respective scores in the Supplementary file -2.

2.      It is important to be careful with terminology throughout this manuscript lest a flippant reader infer a clinical results instead of something else (I would suggest this paper as a guide: PMID: 30962250)
a.      Label ‘nonsense’ or ‘loss-of-function’ instead of ‘pathogenic’ when referring to variant type (e.g. Figure 4d)
b.      Inappropriately using term ‘pathogenic’ instead of the appropriate term ‘non functional’
c.      PIF-based ‘categorization’ should be used instead of PIF-based ‘classification’

We thank the reviewer for their suggestion. We have now revised the entire manuscript based on these guideline.

3.      There seems to be some citations missing:
a.      the splicing effect of p.D2723G

We have included the citation that we had inadvertently missed for the splicing effect of p.D2723G (*reference # 50, Walker et al. Hum. Mut. 2010 and reference # 51, Sanz et al. Clin Can Res. 2010*).

b.      the hypomorphic SNV p.Leu2510P (CTG>CCG)

Our group has previously classified the hypomorphic variant for p.Leu2510P in Biswas et al., *Blood* 2011 and the recent characterization of a genetic mouse model expressing this variant (Mishra et al., *Nature Communications*, 2022).

4.      The discussion around comparing results with previous functional data and other lines of variant interpretation evidence is hard to follow. Please consider including a supplementary table listing the following items:
a.      c. (in HGVS, please)
b.      p. (there is a need to be consistent with either one- or three-letter AA nomenclature)
c.      Exon
d.      Functional Score (DMSO, cisplatin, Olaparib)
e.      PIF
f.      aGVGD score
g.      CADD score
h.      BayesDel score
i.      PRIOR
j.      Previous functional result
k.      ClinVar consensus classification

We have revised the text accordingly. We have included the suggested information in Supplementary File 2

5.      Please specifically address how this assay can make up for shortcomings in other functional studies. For example, as this assay uses integrated BAC, it is capable of discerning a splice defect in addition to a protein defect. It should also include any defects that may result from mislocalization for the same reasons (specifically, this is a missed opportunity refuting the idea around p.P2767S which is purported to have normal HDR activity but may have impaired nuclear localization yet is functional in the current study which should also reflect non-functional for mislocalization).

We agree with the reviewer and acknowledge the potential utility of our BAC integration approach for investigating splicing effects compared to other functional assays. However, studying the effects of splicing for all the variants while interesting is indeed a comprehensive endeavor that extends beyond the focus of our current study.

Regarding p.P2767S variant, we would like to clarify that the impaired localization observed in the flag-tagged-minigene assay does not necessarily reflect the actual behavior of the variant in its physiological context. We acknowledge that truncated/minigene constructs can sometimes affect nuclear entry and impact the interpretation of cellular localization-based assays. In contrast, our humanized-mESC approach, which involves a knock-in of BRCA2, provides a more physiological context for studying the variant’s function and behavior. Based on our analysis, which includes cell survival and sensitivity to DNA damaging drugs, we classified pP2767S variant as neutral. This classification is supported by the normal HDR activity conducted by the Couch laboratory. Additionally, we utilized SpliceAI tool to assess the potential splicing effects of this variant and didn’t identify any spliced forms that may affect its function.

It is crucial to consider the potential differences that can arise due to cell line and assay-specific factors. By integrating multiple lines of evidence and considering the strengths and limitations of different approaches, we aim to provide a comprehensive understanding of variant function and categorization. We have incorporated these clarifications into the revised manuscript. (Page 16, sentences 1-4)

6.      Broadly, I understand that this is probably a proof-of-principle study that is likely paving the way for a larger study. However, I think this paper falls short in the validation methodology, not only because of the small number of clinically observed/classified variants, but also because it is not well connected to the clinical interpretation of the variants. While it is certainly worthwhile to compare the functional result to in silico predictors, previous functional data, and ClinVar assertions, there is no hard-and-fast classification truth set. ClinVar is a bit of repository free-for-all and it is difficult to tap credible classifications (also their caller for ‘conflicting’ or ‘consensus’ classifications is rather bad). The best approach would be to partner with a credible clinical lab (or two or three). One critical piece would be to look at the clinical interpretation from a cumulative body of classification evidence that excludes functional data, since using
functional data in the final classification would be a bit circular in light of validating another functional study (functional studies are not often not truly agnostic and are measuring the same outcome with a different read-out). The other option would be to use ClinVar judiciously so only credible calls are considered in this comparison but omitting functional data from a final interpretation would not be easy in this

We appreciate the reviewer’s recognition of the proof-of-principle nature of our study and the importance of future applications of SGE for the entire BRCA2 gene. We agree that *in silico* predictions should be interpreted with caution and that our study goes beyond relying solely on computational meta predictors. To ensure the reliability of our findings, we conducted extensive validation using various functional assays developed by other laboratories and using different cell lines. These include the HDR-based assay from the Couch lab, drug sensitivity assays from the Mano lab, and consideration of ClinVar predictions. Additionally, many of the variants analyzed in our study, such as p.T2722R, p.D2723G, p.D2723H, and others, have been studied by many laboratories, including population-based studies. The strong overlap between our data and previous findings further supports the robustness of our results.

We agree with the reviewer to consult with clinical labs and such collaboration with clinical labs would provide valuable insights and enable a comprehensive analysis of the functional consequences of BRCA2 variants. However, initiating such collaborative effort is beyond the scope of the current study, which we would consider in the large-scale functional study encompassing all possible variants of BRCA2 in future.

7.      Could the authors please comment on the striking number of non functional variants in this dataset (more than half!), e.g. figure 4d with 313 non functional variants, 278 functional variants. This is way more than what is normally expected from the variation observed in the whole genome of which most of it is benign.

a.      I suspect this is a reflection of the specific codons that were selected but the authors do not comment on the rationale for selecting these specific exons/codons. Were they selected because they are known to be sensitive to missense variation based on observed LP/P variants in the literature/databases? Surely they aren’t really random!

We thank the reviewer for giving this opportunity to discuss this important point. Since the current study is a saturation genome editing approach targeting single and multi-nucleotide variants, it is possible to generate more non-functional variants. For example, changing residue Asp at 2723 to Asn, Tyr, Ala, Gly and Val all considered to be non-functional, as previously reported. In addition, changing Asp to Val and Glu is also non-functional. Hence, we believe this reflects the specific residues used in this assay and the reason to have more nonfunctional variants in this assay. We agree with the reviewer and believe that Saturation genome editing (SGE) to all the possible single-nucleotide variants will lead to more variants that are benign and some of our ongoing preliminary data also suggests the same.

As this study is aimed to establish SGE in BRCA2, we chose to target specific residues across a few exons that have been well-characterized by previous functional assays and population data. The inclusion of known non-functional variants such as p.Thr2722Arg (T2722R) and p.Asp2723His (D2723H) in exon 18 was deliberate. These variants have been studied by multiple laboratories and serve as important controls in our assay. By including these well-known non-functional variants, we can validate the performance of our SGE approach and ensure its accuracy and reliability. We have clarified the rationale behind our selection of specific residues and the goal of establishing SGE in BRCA2 in the revised manuscript.(Page 6, 2^nd^ paragraph).

8.      “Currently, a non-functional variant can be distinguished from a functional variant based on its co-segregation with the disease within families, as well as its frequency in the control population”: this sentence is incomplete in terms of the lines of evidence that can be used to classify variants (presuming the use of ‘non-functional’ and ‘functional’ here actually mean ‘pathogenic’ and ‘benign’). Rework this and cite Richards 2015 PMID 25741868.

We have revised the sentence and cited the standards and guidelines outlined in Richards et al. 2015. (Page-4, 3^rd^ sentence).

9.      In general, the figures need to be updated throughout to have the same scale on the y-axis (this includes supplementary files) particularly when trying to draw comparisons between conditions where distributions are displayed.

a.      E.g. Supplementary figure 4d, at first glance, there appears to be no change in the distribution of variant classes when exposed to cisplatin and Olaparib. However this is probably because the scale changes from the DMSO plot (goes up to 60) and the drug plots (go up to 40): the effect might be better observed if the scale was to 60 for every plot here.

We appreciate the reviewer’s feedback regarding the figures and have updated them throughout the manuscript. This ensured consistent scaling, allowing for easier comparison and analysis of the data.

10.     It is not clear to this reader why part of the figures are parsed by which exon the data originates while others ignore this altogether. E.g. Supplementary figure 2b and d and Main figure 2b are all about which exon things are in. But moving on to figure 2c, e, and f, this concept is lost. Is the exon relevant or not? It seems to not-be discussed much in the text, but I do think it’s interesting given what we know about functional domains for BRCA2.

We agree with the reviewer that highlighting the exon-specific information is important to provide a detailed understanding of the functional impact of variants in different regions of the BRCA2 gene. Throughout the manuscript, we have made efforts to present the data in a manner that captures both the exon-specific details and the overall distribution of functional scores.

Apart from Supp. fig 2b, d, the main fig-2b; fig. 2d, 3E are also presented as exon specific data. However, we think data represented in supp. fig. 3 and 4 summarize the overall distribution and trends observed across the entire dataset. The individual function scores are shown in main fig. 2d. We have added this information in the text corresponding to Fig.2d.

The statistical model is built by taking the entire dataset into consideration, and separating the PIFs by exons may not be the most appropriate approach for this analysis. The sensitivity and specificity of statistical models are better when large number of variants and several known benign, pathogenic controls are annotated in a dataset. This is also relevant as our future aim is to analyze all possible mutations across *BRCA2*. However, we have provided the individual function scores data as a supplementary file, allowing readers to explore the exon-specific analyses if desired.

11.     In Figure 4d, there appears to be substantial overlap in the PIF functional and PIF non functional categories around a functional score of 0 to -2. Shouldn’t this be avoided by adjusting the model so these are all intermediate for better specificity of the caller?

We thank the reviewer for this very valid point. We have indeed adjusted the statistical model to include the scores from the DMSO, cisplatin and olaparib assays. As described on pages 10-12, we have demonstrated that this three-variable model successfully classifies variants into functional and non-functional categories with minimal overlap, except for a small number of variants that fall into the intermediate/indeterminate category.

Furthermore, we have provided evidence to support the superiority of the full three-variable model over the one-variable models, as shown in Supp. Fig. 3A-C**.** The one-variable models resulted in a considerably larger number of variants falling into the intermediate category, leading to higher prediction ambiguity. This emphasizes the advantage of including all three variables in the statistical model, as it significantly improves the performance and reliability of the classification. This comprehensive approach provides a more robust and accurate classification of BRCA2 variants, allowing us to assess their functional impact with greater confidence.

12.     Figure 1. Although correctly labeled in the text, it would be best to relabel this figure so the boxed confirmatory tests itemizations match the figure label. For example: in the box, b. Southern blot | c. qPCR |d. Western blot, etc.

We thank the reviewer for their suggestion and have now revised Fig.1 to incorporate these changes.

13.     Figure 1C what is the significance of the lanes labeled F7 and F11…are these two clones? I know KO and cko represent the knockout and conditional knock out but you should probably put those abbreviations in the legend.

F7 and F11 are two clones and F7 clone showed higher intensity of the *BRCA2* Transgene, hence used for downstream experiments. We have added this information in the figure legend. We have now explained in fig.1b what KO and CKO represent.

14.     Supplementary Figure 2b: It’s interesting that there is more variability for exon 18 variants than the other two (perhaps because there are more non-functional variants?). Could the authors please comment and also produce a statistic for each exon independently as well as grouped?

We have added the statistic for each exon and based on spearman’s correlation, we observed a good correlation in read counts between the replicates for each exon. Please refer to revised Supp. fig. 2b.

15.     Figure 2b and Supplementary Figure 2d: could the authors please explain why there is no difference in the day 3 and day 14 replicates for exon 3? As indels (mostly frameshifts), these should be equally subject to NMD (and reflect non-functional (read depletion)) in exon 3 as in exons 18 and 19, but that is clearly not the case.

We acknowledge the reviewer’s comment regarding the persistence of variants with frameshift indels. While we do not precisely understand how variants with a single nucleotide deletion are able to persist, it is worth noting that the majority of out-of-frame indels exhibit a significant fitness impact. Additionally, there is a decrease in the occurrence of stop codons from day 3 to day 14, and synonymous variants are present in the variant pool. These observations suggest that the functional interpretation of the variants in this exon remains largely unaffected.

16.     Supp fig 4a,b,c please label the colors for the dots in the legend or on the figure

We have now included information on color labels in the figures which shows that Blue is Functional, Red is Nonfunctional and Gray is intermediate.

17.     Figure 3e: could the authors please find a way to label these with the p. since the text references the p. throughout. One idea I had is to replace the © indicating tested from other assay with the actual one-letter protein code (keeping with the asterisk formatting for nonsense) and indicate the ‘tested from other assay’ as a superscript asterisk/other character. It would also be good to indicate which variant(s) were described as having a splice effect (perhaps a different superscript character). I would avoid using the X to indicate ‘no data’ as it seems like a stop codon in some nomenclature. Maybe just choose a dash instead or leave it black or gray). Overall-this figure could use a bit of polish (e.g alignments are off and circles are different sizes)

We thank the reviewer for their thoughtful suggestions to improve our data visualization. We have now revised fig.3e as per the reviewer’s suggestions. Specifically, we have added the actual one letter protein code to keep it consistent with our main text. The variants “tested from other assay” are denoted by an outer circle. The variants with “no data” are represented by a black box.

18.     Figure 3c/d. Why are there different number of SNVs in the CADD group and the BayesDel group? There is a label for gray intermediate variants but I see none on these plots.

In our initial analysis, we had used the BayesDel scores derived directly from the author/creator of the score ([https://fengbj-laboratory.org/BayesDel/BayesDel.html](https://gcc02.safelinks.protection.outlook.com/?url=https%3A%2F%2Ffengbj-laboratory.org%2FBayesDel%2FBayesDel.html&data=05%7C01%7Csounak.sahu%40nih.gov%7C0894beb180774d69fefe08db67c3c941%7C14b77578977342d58507251ca2dc2b06%7C0%7C0%7C638217862819870104%7CUnknown%7CTWFpbGZsb3d8eyJWIjoiMC4wLjAwMDAiLCJQIjoiV2luMzIiLCJBTiI6Ik1haWwiLCJXVCI6Mn0%3D%7C3000%7C%7C%7C&sdata=ZWv4ftGrABS6T%2FfQD39VDqiIOzbghVDx6b4unClaI6Q%3D&reserved=0)). However, the scores and the annotation software used have not been updated since 2017 (and the scores were built with the hg19 genome build. This original build was missing some variants likely due to the incomplete lift over process from hg19 to hg38. We have updated the BayesDel scores derived using ANNOVAR (Version 2020-06-08), from the dbNSFP v4.3 database which incorporates the BayesDel and other annotation scoring methods.

We have revised the plots for 71 SNVs for CADD and Bayes-del scores in revised supp. fig S5. CADD and Bayes-Del do not provide scores for synonymous variants; hence they were excluded. We do not have any intermediate SNVs and hence not visible in the plots. We have removed the gray dot from the figure.
