# Supplementary material for: Saturation genome editing of 11 codons and exon 13 of BRCA2 coupled with chemotherapeutic drug response accurately determines pathogenicity of variants
Source: PLoS Genet. 2023 Sep 15;19(9):e1010940. doi: 10.1371/journal.pgen.1010940 (PMC10529611; doi:10.1371/journal.pgen.1010940)
Supplement: Attachment — Submitted filename: Sahu et al_Response to Comments.docx [file pgen.1010940.s012.docx]

**Point by point response to reviewer’s comments (PGENETICS-D-23-00242R1)**

We sincerely thank the reviewers for their valuable feedback. We have addressed their concerns and revised the manuscript based on the suggestions as described below.

**Reviewer #2:** This revised manuscript from Sahu and colleagues is improved and addresses most of my concerns. In particular, adding the full exon 13 SGE goes a long way towards demonstrating the scalability of the approach.

We appreciate the reviewer’s acknowledgement of the improvements made in the revised manuscript. We are pleased to see that the inclusion of the full exon 13 SGE has contributed to showcasing the scalability of our approach.

To preview the road ahead, the authors should also include a rough estimate as to the number of experiments required to flesh out the whole gene, or at least however much of it they feel has sufficient pathogenic missense burden to justify the work.

Based on our findings in this manuscript, we propose that a region spanning 50-100 nucleotides can effectively be saturated using a pair of sgRNAs. This suggest that any region of BRCA2 could potentially be saturated, contingent on the availability of sgRNAs at intervals of 30-50 nucleotides. For instance, the C-terminal DNA binding domain of BRCA2, encoded by exon 15 to 26 and spanning 2213 nucleotides, hosts many pathogenic missense variants. We predict that this 2200 bp region could be comprehensively saturated for all possible single nucleotide variants (SNVs) using approximately 50 oligo donor libraries in conjunction with a single or a pair of sgRNAs. We have now mentioned this in the revised manuscript on pages 21-22.

My major remaining concern is around the claims that the assay can identify partial loss of function mutations using DNA damaging agents. (“BRCA2 variants with a loss of function are expected to be depleted … and can be regarded as pathogenic, whereas variants with a partial loss of function may not be significantly depleted by day 14. However, these latter variants are expected to exhibit sensitivity to DNA-damaging drugs”). This portion of the manuscript remains poorly supported. I do not see any evidence that the drug treatments are any adding information beyond that provided by the DMSO treatment, for the following reasons: (1) Nonsense-synonymous separation seems to be nearly identical between DMSO, cisplatin, and Olaparib. (2) In the plots provided, DMSO-DMSO replicate difference seems comparable to that of either compound versus the other or versus DMSO (3) That one-condition PIF models outperform the full model probably just reflect the fact that there are more replicates in the latter.
Moreover, the “NNN” codons provide an opportunity for internal replication by virtue of multiple codons encoding for the amino acid change; for the examples of intermediate effect the authors highlight, these point to the explanation being an averaging of noisy data rather than a true intermediate/hypomorphic effect. For instance, an example is given of p.Glu58Val (c.173_174AA>TG), which in Table S3 is listed as having a final PIP score of 0.278 (note—not less than 0.2 as the manuscript states). The other three equivalent codon swaps (GAA>GTA,GTC, and GTT) all have PIP scores of ~0, and are classified as functional.
I think the manuscript would be stronger without the claims about intermediate variants, but if included, they should be better supported.

We thank the reviewer for their suggestion and thoughtful insights. We have taken their input seriously and have made significant revisions to the manuscript to address this concern. In the revised version of the manuscript, we have provided a clear explanation regarding the classification of variants based on PIFs falling within the range of 0.05 to 0.99 in our assay.

Furthermore, we have clarified that variants falling within this range are considered “intermediate”. This categorization signifies that our assay does not definitively classify these variants as either functional or non-functional based on the data obtained. It is important to note that this interpretation of “intermediate” does not inherently imply a hypomorphic nature of the BRCA2 variants.

We acknowledge the reviewer’s astute observation that further analysis is required to comprehend the potential hypomorphic characteristics of variants falling within this intermediate range. While our assay aids in identifying variants that are either functionally disrupted or retained, additional investigations are necessary to ascertain if variants in the intermediate range indeed exhibit hypomorphic properties. This understanding will contribute to a more comprehensive assessment of the functional impact of these variants. We have clarified this point on page 13.

Minor
- Figure legends are really on the long side

As suggested by the reviewer, we have reduced the length of the figure legends in the revised manuscript. Specifically, in Figure 2, we have moved extensive details about the PIF classification to the methods section.

- “Humanized HEK293T cells” should be “Haploidized HEK293T cells” (it is a human cell line)

We apologize for the oversight in our revision. We have rectified the error and changed it to “haploidized”.

- Terminology throughout is still confusing and a bit nonstandard. In several places “non-functional” and “functional” are used to mean “nonsense” and “synonymous”, which as the more specific and descriptive terms should be used. “Experimental” seems to be used to refer to all other elements of the library, which is similarly vague; how about “library” or “missense library” for that?

We appreciate the reviewer’s insightful comments regarding the terminology used in the manuscript. To specifically address the categorization of variants, we now refer to variants generating stop codons as “nonsense” and variants with no change in amino acids as “synonymous”. We believe presenting variants as “missense library” could be misleading, as synonymous and nonsense variants are also present in the same oligo library. Therefore, to provide a more distinct and comprehensive categorization, we have labeled all other variants as “experimental”. This will eliminate any potential confusion and ensure that readers can clearly understand the nature of each variant. We have included this information in the revised manuscript, please see legend for Figure 2e.
“We found a strong correlation… between the two independent replicates across exon 3, 18, and 9” (should 9 be 19 here?)

Thanks for pointing this; we have now corrected this typo.

**Reviewer #3:** The authors have done considerable work to address a number of comments, and the manuscript is substantially improved. In addition, expansion of the assay across exon 13 is a considerable improvement to show that this is a significant approach.

We appreciate the reviewer’s acknowledgement of our efforts in addressing the comments and improving the manuscript. We are particularly pleased to know that the expansion of the assay across entire exon 13 has been seen as a meaningful enhancement. We thank the reviewer for their valuable feedback and address their remaining concerns as follows.

However. there are still some points that need addressing, some relating to the original questions, and some due to additional edits. The grammar in the first sentence of the abstract still needs work. Two grammatical errors remain.

We have revised the first sentence of the abstract. “*The unknown pathogenicity of a significant number of variants found in cancer-related genes is attributed to limited epidemiological data, resulting in their classification as variant of uncertain significance (VUS).”*

The new distribution plots in Fig 2c are in improvement and do show that there is overlap in the scores for synonymous and stop codon variants, albeit minimal. **Could the authors include an F test to confirm normal distribution.** And would it be possible to generate a plot similar to Fig 2c where the PIF based on all three assays is displayed only for the synonymous and stop codon variants, in order to show separation of PIF for these variant as determined based on the three scores combined.

We have performed F-test to confirm the normality of the variant distribution and added the data in the legend of Figure 2. The blue (synonymous variants) and the red (nonsense variants) circles in Figure. 2e show the separation of the PIF score that is determined based on the three scores combined.

Fig 2d – thank-you for the correction. Could the fig 2 title also be amended eg change “to classify” to “to inform classification of”

Thank you for pointing this. We have revised the title of figure 2 and changed it to “to inform classification of”.

The authors have added supplementary file 2 but appear to have not even referred to it in the amended text.

We regret that we inadvertently missed to refer supplementary file 2 in the revised text. We have now referred to this on pages 15, 20 and 29.

More discussion is required, including a discussion of how variants/results overlap with what was included in training dataset in addition to the synonymous and stop codon variants.

We have now discussed the variants included in the training dataset on page 20.

Additional comments relating to calibration and clinical evidence strength calculation.
The new manuscript page 30 (page 59 of the document), methods section 9, states “We calculated positive Likelyhood Ratio (LR+) and negative Likelyhood Ratio (LR-) to provide an indication of the probability of a variant being pathogenic based on our functional assay results.”
First - likelyhood ratio should read “likelihood ratio”.

We apologize for the typo, which we have now corrected. We have changed "likelyhood ratio” to “likelihood ratio”.

Regarding the calculation for positive LR and negative LR – as written the calculations seem to assume that there is no grey zone – no region where the functional impact is unclear. Based on results shown in fig 2c, there would be value in assigning a zone of functional score as “unclear”, where both synonymous and stop codon variants overlap in functional score. Could the authors confirm if only the synonymous and stop codon variants were used to define this sensitivity and specificity? Or were the clinvar LP and PB variants used as well (see below). If so then those variants should also be plotted in Fig 2c and any plot that shows the PIF based on all three scores.

The data presented in Figure 2C corresponds to the synonymous and nonsense variants identified from the proof-of-principle approach to establish SGE of BRCA2 for 11 codons across exon 3,18 and 19.

The authors state that the Brnich calibration method cannot be used, as this requires multiple lines of evidence. There appears to be some confusion since the authors could use previously classified variants (eg those in clinvar) as controls to calibrate. Indeed they might have done this, as per this text in the methods: “All non-sense variants and all variants classified as “Pathogenic” or “Likely pathogenic” in ClinVar database constituted the “Non-functional” class in the training dataset. All synonymous and all variants classified as “Benign” or “Likely benign” in ClinVar database constituted the “Functional” class. 42 known functional and 12 known non-functional variants were used for the training dataset.”
Could the authors please clarify where these numbers come from, and how they do or do not overlap with the numbers in figure 2c (21 synonymous and 29 stop codon).

We thank the reviewer for bringing up this important point. We regret any confusion caused by our previous statements. The clarification regarding the potential use of previously classified variants as controls for calibration is indeed valid. In our manuscript, we utilized previously classified variants that are reported in ClinVar database for the training dataset in Exon 13 SGE. This training dataset corresponds to 42 known “functional” corresponding to ClinVar classified “Benign” and “likely benign” variants and 12 known “non-functional” corresponding to ClinVar classified “Pathogenic” and “likely pathogenic” variants. These variants are distinct from the data provided in fig 2c corresponding to 21 synonymous and 29 nonsense variants collected from the proof-of-concept study to classify 599 variants across 11 codons from Exon 3, 18,19. We have included this information on pages 27 and 31.

A reasonable approach would be to use the synonymous and stop codon variants to set the original cutpoints for what is functional, not functional and intermediate (where scores are overlapping, and then formally calibrate the functional assay categories in an independent set of variants of known pathogenicity from ClinVar. Obviously, it would be important to assess if these variants could be classified without use of other functional data, but it should be possible. That would add value for interpretation of findings since the application of the Gaussian mixture model is not inherently intuitive to curators and would allow simple application of LR cutoffs determined for weighting ACMG codes.

We appreciate the reviewer’s recommendations for enhancing the interpretability and clinical relevance of our study. The suggestion of utilizing synonymous and nonsense variants to establish initial cut points, resonates well with our goals. As an additional approach we estimated the evidence strength of our assay using 43 synonymous and 8 non-sense SNVs from exon 13 as the training dataset and our model exhibited 85.7% sensitivity and 100% specificity in classifying ClinVar reported variants. Please see revised Supp. fig. 6h.

Given that the authors are stressing the potential clinical importance of their study, doing the additional legwork to calibrate using the Brnich* approach would seem to be in scope and a lot easier than doing extra functional assays. That is, please calculate the diagnostic odds of pathogenicity for the assay based on a reference/calibration set that does not overlap with the original set of variants use to set the thresholds.

*Brnich, S.E., Abou Tayoun, A.N., Couch, F.J. et al. Recommendations for application of the functional evidence PS3/BS3 criterion using the ACMG/AMP sequence variant interpretation framework. Genome Med 12, 3 (2020). <https://doi.org/10.1186/s13073-019-0690-2>.

We acknowledge the suggestion to calculate the OddsPath value based on a reference set that is distinct from the original variant set. We agree that this strategy will add to the clinical implications of our work and have now incorporated this analysis in the revised manuscript. Following the ACMG guidelines, the Odds of Pathogenicity (OddsPath) value of our model is 9.52 for pathogenic supporting PS3_moderate level of evidence and 0.06 for benign variants supporting BS3_moderate level of evidence. Brnich et al. recommends Moderate level evidence in favor of pathogenicity (PS3_moderate) or benign interpretation (BS3_moderate) for assays with at least 11 total validation controls including a mix of benign and pathogenic variants. We have 27 benign/likely benign and 17 pathogenic/likely pathogenic variants from ClinVar. For training dataset, we have 43 synonymous and 8 non-sense SNVs. We have mentioned this in the revised manuscript on page 20.

Splicing - The manuscript has added in information regarding the consideration of splice variants. Noting that the SpliceAI results have been added to Supp File 4, it is not clear how these SpliceAI results were used to inform potential impact on splicing. Specifically, the authors state “Furthermore, sgRNAs were selected based on SpliceAI predictions such that the synonymous PAM mutation does not affect canonical splicing”. It appears in the table that there are SpliceAI scores well above 0.2. Could the authors please clarify exactly what cutpoints they used to assess potential impact on splicing. A recent calibration experiment arising from a ClinGen group (<https://doi.org/10.1016/j.ajhg.2023.06.002>) recommends conservative cutpoint of 0.2 to indicate potential splicing impact. The data provided in Supp File 4, at least at a quick glance, would suggest that some of the variants have potential to alter splicing… If all of these were included as PAM alterations, then this this could have implications for interpretation of the functional assay findings

We appreciate the reviewer’s examination of the information provided regarding the impact of splice variants and the integration of SpliceAI results. We realize that our initial explanation may have left room for misunderstanding, and we thank you for bringing this to our attention. We concur that the conservative cutoff of 0.2, recommended by the recent calibration experiment (reference #43), as mentioned by the reviewer, is a valuable benchmark for indicating potential splicing impact. We have mentioned this in the revised methods section on page 33.

Upon revisiting our data and based on the reviewer’s concerns, we have highlighted the variants that have splice AI >0.2 and have revised Figures 3 and 4. We have included a blue box for the variants that are predicted to affect splicing based on SpliceAI results. Majority of these variants are classified as non-functional in our assay. We would like to clarify that the presence of PAM mutation alone does not impact splicing using the SpliceAI score cut off = 0.2. We have also included this information in supp. file 4.

Additional comment. There still appears to be ambiguity in the difference between pathogenic (as in a validated, curated, clinical disease associated variant) and one that scores as loss of function as per assay results from this study.

We appreciate the reviewer’s observation and understand the importance of clarifying the distinction between disease associated pathogenic variants and those identified as loss of function (LOF) based on our assay results. We apologize for any confusion that may have arisen due to this ambiguity.

We acknowledge that these function scores are not binary, and they indeed reflect a spectrum of functional consequences that may be influenced by various factors, including protein stability, half-life, and cellular context. Our proposed Probability of impact on function (PIF) metric aims to capture the likelihood of a variant causing a functional impact, rather than assigning a binary outcome. We agree that this approach aligns with the findings of previous studies that have employed high-throughput deep mutational scanning to explore the functional consequences of variants across different genes.

By using the PIF metric, we intend to provide a more comprehensive representation of the potential functional impact of variants, considering the complexity of protein function and its interplay with cellular processes. We believe that this approach enhances the accuracy of our assessment and provides a more nuanced understanding of the variants’ effects.
